# Deep Learning-Based Synthetic Contrast-Enhanced Breast MRI for Monitoring Response to Neoadjuvant Therapy

**DOI:** 10.3390/cancers18111835

**Published:** 2026-06-04

**Authors:** Suleeporn Sujichantararat, Debosmita Biswas, Anum S. Kazerouni, Edric D. Tsang, Aditi Sathe, Daniel S. Hippe, Vivian Y. Park, Maggie Chung, Jennifer M. Specht, Suzanne M. Dintzis, Habib Rahbar, James H. Holmes, Wei Huang, Savannah C. Partridge

**Affiliations:** 1 Department of Radiology, University of Washington, Seattle, WA 98195, USA; ssuji@uw.edu (S.S.); biswasd@uw.edu (D.B.); anumkaz@uw.edu (A.S.K.); etsang09@uw.edu (E.D.T.); aditis05@uw.edu (A.S.); hrahbar@uw.edu (H.R.); 2 Department of Bioengineering, University of Washington, Seattle, WA 98195, USA; 3 Clinical Research Division, Fred Hutchinson Cancer Center, Seattle, WA 98109, USA; dhippe2@fredhutch.org; 4 Department of Radiology and Research Institute of Radiological Science, Yonsei University College of Medicine, Seoul 03722, Republic of Korea; vivianpark0116@yuhs.ac; 5 Department of Radiology and Biomedical Imaging, University of California San Francisco, San Francisco, CA 94143, USA; maggie.chung@ucsf.edu; 6 Division of Hematology and Oncology, University of Washington, Seattle, WA 98195, USA; jspecht@uw.edu; 7 Department of Laboratory Medicine and Pathology, University of Washington, Seattle, WA 98195, USA; dintzis@uw.edu; 8 Departments of Radiology, Biomedical Engineering, and Electrical and Computer Engineering, University of Iowa, Iowa City, IA 52242, USA; jim-holmes@uiowa.edu; 9 Department of Radiation Oncology, Research Institute, Corewell Health, Royal Oak, MI 48073, USA; wei.huang@corewellhealth.org; 10 Department of Radiation Oncology, Oakland University William Beaumont School of Medicine, Rochester, MI 48309, USA

**Keywords:** breast, cancer, gadolinium, treatment response, MRI, neoadjuvant therapy (NAT), residual cancer burden (RCB), pathologic complete response (pCR), synthetic contrast-enhanced MRI modeling

## Abstract

**Background/Objectives**: Contrast-enhanced (CE) breast MRI is highly sensitive for evaluating breast cancer extent and response to neoadjuvant therapy (NAT) but requires intravenous administration of gadolinium-based contrast agents (GBCA), increasing cost, time, patient discomfort, and health concerns. This study explored the feasibility of reducing GBCA use in treatment monitoring using a deep learning (DL) model to synthesize CE-MRI from non-contrast MRI. **Methods**: This IRB-approved retrospective pilot study evaluated women with breast cancer enrolled in an ongoing trial using serial MRI to monitor NAT prior to surgery. A pre-trained DL model was used to synthesize CE-MRI from T1-, T2-, and diffusion-weighted MRI. Changes in tumor volume at early (post-1-cycle NAT) and mid-treatment were measured on synthetic and acquired CE-MRI. Performance for predicting residual cancer burden (RCB) class 0/1 was evaluated using AUC and compared with DeLong’s test. **Results**: 27 women were included in the study (median age, 47 years [range = 28–75]); 14 (52%) achieved RCB class 0 and six (22%) achieved class 1. Synthetic CE-MRI-derived tumor volumes showed strong correlation with those from acquired CE-MRI at pre-treatment (ρ = 0.92, *p* < 0.001) and early treatment (ρ = 0.83, *p* < 0.001), but lower agreement at mid-treatment (ρ = 0.57, *p* = 0.002). Change in tumor volume on synthetic CE-MRI was numerically similar to acquired CE-MRI for predicting RCB class 0/1 vs. 2/3 at both early (AUC = 0.84 vs. 0.86, *p* = 0.83) and mid-treatment (AUC = 0.73 vs. 0.75, *p* = 0.80). **Conclusions**: Synthetic CE-MRI demonstrates preliminary feasibility as a non-contrast surrogate for predicting favorable outcomes (RCB class 0/1) in this pilot study, but inconsistencies in tumor volume measurement vs. acquired CE-MRI warrant further model refinement and validation.

## Simple Summary

Contrast-enhanced (CE) breast MRI is a highly sensitive technique commonly used to monitor breast cancer treatment response. CE-MRI requires intravenous administration of gadolinium-based contrast agents (GBCA) by trained medical professionals, which is costly, time-consuming, and adds to patient discomfort and health concerns. Emerging artificial intelligence models hold potential to reduce GBCA use by synthesizing CE-MRI from non-contrast MR images. This proof-of-concept pilot study aimed to evaluate the effectiveness of synthesized CE-MRI in measuring tumor volumes and monitoring response to neoadjuvant therapy (NAT). Changes in tumor volume calculated at early treatment (post-1-cycle NAT) and mid-treatment were used to predict final pathological response outcomes. Results showed that synthetic CE-MRI provided numerically similar predictive value to acquired CE-MRI, demonstrating its preliminary feasibility as an early marker of response, while also suggesting the need for further refinement of the model to accurately capture residual tumor volumes and validation in larger and more heterogeneous cohorts.

## 1. Introduction

Breast cancer is the second-highest cause of cancer-related death among women in the US [1], and timely detection and treatment can save lives. Neoadjuvant (or preoperative) therapy (NAT) is a common approach to treating breast cancer patients due to its benefits in decreasing tumor size, enabling breast conservation surgery, forecasting prognosis, and guiding personalized adjuvant therapies [2]. However, some patients derive minimal benefit from the treatment while still having to endure its toxic effects [3]. This underscores the need for early markers of treatment response that support more personalized and effective treatment. Such biomarkers have potential clinical utility in various aspects of multidisciplinary treatment planning, where breast surgeons and clinicians could use improved early response characterization to better stratify patients to optimal treatments and inform surgical decision-making after NAT. In particular, more accurate and earlier identification of robust response to NAT may eventually contribute to treatment adaptation strategies, including potential de-escalation of surgical management and increased consideration of breast-conserving approaches in appropriately selected patients [4,5,6,7], although these applications remain investigational and require further validation.

Monitoring response to NAT typically relies on physical examination and serial imaging to track changes in tumor size [8]. Pathological response to NAT, based on pathology review of surgical specimens, is an established predictor of survival outcomes [9]. Contrast-enhanced (CE) magnetic resonance imaging (MRI) is commonly used for NAT monitoring in patients with invasive breast cancer, and a change in tumor volume on CE-MRI has been proven as an effective imaging biomarker for early prediction of both pathological response and long-term treatment outcomes [10,11,12,13].

CE-MRI relies on intravenous injection of gadolinium-based contrast agents (GBCAs) to highlight areas of abnormal tissue vascularity, aiding physicians in identifying breast cancer and assessing treatment response. However, the need for GBCAs is undesirable for multiple reasons (time, costs, patient discomfort, and health concerns) [14,15] and can limit accessibility. Challenges of CE-MRI include lengthy examination time due to venipuncture setup and dynamic contrast imaging sequences, increased healthcare costs related to contrast agent use and extended scanner time, and the need for post-injection monitoring. Although acute severe contrast reactions are rare, they can occur, and concerns regarding gadolinium retention in the body have been reported. While newer, more stable gadolinium agents that are less likely to release gadolinium into the body are considered safer, some patients remain hesitant to receive GBCA injections due to perceived, though not fully established, long-term health risks, which may limit CE-MRI acceptance in certain populations. Toward the goal of reducing the need for GBCAs, multiple studies have utilized deep learning (DL) to synthesize CE-MR images from non-contrast MR images and shown promising value for breast cancer evaluation [16,17,18,19,20]. However, prior studies have primarily focused on image-generation quality and image-similarity metrics, while limited evidence exists regarding whether synthetic CE-MRI preserves clinically meaningful information for downstream applications. In particular, there has not yet been any study exploring the potential of utilizing synthetic CE-MRI for contrast-free monitoring of treatment response.

A synthetic breast CE-MRI DL model developed by Chung et al. [16] is one of the earliest published models to incorporate diffusion-weighted imaging (DWI). The model uses a U-Net backbone, a well-established and widely used DL architecture for medical image segmentation [21] that has also demonstrated effectiveness in MR image synthesis tasks, including applications in brain imaging [22]. The trained model leverages multiple non-contrast MRI sequences, including T1-weighted images without and with fat suppression, fat-suppressed T2-weighted images, DWIs (b = 0 and 600 s/mm^2^), and apparent diffusion coefficient maps, thereby utilizing complementary anatomical and functional information available in a typical multi-parametric breast MRI protocol. By integrating these diverse imaging inputs, Chung et al.’s model provides a promising framework for synthesizing CE-MRI from non-contrast MRI and enabling downstream applications such as treatment response assessment.

Therefore, the objective of this study was to evaluate the utility of this previously developed synthetic CE-MRI DL model [16] to measure changes in tumor volume and predict breast cancer response to NAT. Specifically, we investigated the following research questions: (1) whether tumor volume measurements derived from synthetic CE-MRI demonstrate agreement with acquired CE-MRI across serial NAT examinations, and (2) whether synthetic CE-MRI preserves sufficient quantitative information to support early prediction of pathological treatment response.

## 2. Materials and Methods

### 2.1. Patient Population

This retrospective study utilized data from women with breast cancer enrolled in an ongoing IRB-approved trial using serial MRI for monitoring NAT prior to surgery (NCT05704062). MRIs were performed at 4 time points before surgery: pre-treatment, early treatment (after one cycle of NAT), mid-treatment, and post-NAT (prior to surgery). Eligible study patients included 31 women enrolled at our institution in the ongoing trial between October 2022 and February 2025 who underwent serial MRI to monitor NAT response prior to surgery. After excluding patients with implants (*n* = 1), MRI artifacts (*n* = 1), and non-mass enhancement (NME) as the primary lesion type at pre-treatment (*n* = 2), a total of 27 women were included in the study cohort. Patients with NME lesions were excluded because the synthetic CE-MRI model had primarily been characterized for mass-type lesions. This study included MRI data from baseline through mid-treatment time points to evaluate the potential for early response prediction. One patient did not undergo mid-treatment MRI, resulting in missing data for that time point. Accordingly, mid-treatment analyses included 26 patients.

Response to NAT was assessed based on residual cancer burden (RCB) determined by pathological analysis of surgical specimens [23], which evaluates the primary breast tumor and axillary lymph nodes. RCB is generally reported as four classes in the standard of care, including 0 (pathologic complete response [pCR]), where no residual invasive cancer is present, 1 (minimal burden), 2 (moderate burden), and 3 (extensive burden), where a large amount of residual invasive cancer is present. RCB classes 0 and 1 are considered good responses and are associated with favorable treatment outcomes [23].

### 2.2. Deep Learning Model to Synthesize CE-MRIs

Synthetic CE-MRIs were generated using a previously developed DL model [16], for which the trained model parameters were made publicly available by the original authors. The model is based on a U-Net architecture [21], a convolutional neural network originally developed for biomedical image segmentation. As previously described [16], the model was trained on a separate breast MRI dataset (96 exams from 96 patients; not a part of this study) from a separate single-institution dataset independent of the current study cohort to synthesize early-phase post-contrast CE-MRI (approximately 2 min after GBCA injection) from six non-contrast MRI input images, including T1-weighted images without and with fat suppression, fat-suppressed T2-weighted images, DWIs (b = 0 and 600 s/mm^2^), and apparent diffusion coefficient maps (T1, T1FS, T2, DWI_b0, DWI_b600, and ADC maps, respectively). Of note, characterization of the model performance focused primarily on mass-type lesions, with authors acknowledging that more work is needed for accurately evaluating NME.

In the present study, the single fixed externally pre-trained model was applied directly to our dataset after appropriate preprocessing in an inference-only setting, without cohort-specific optimization. All six required input image types were provided to generate synthetic CE-MRIs, and no additional training or fine-tuning was performed. Therefore, potential differences in MRI acquisition protocols, scanner characteristics, and institutional imaging practices between the original training dataset and the current cohort could contribute to domain shift affecting model performance. Details of acquisition and preprocessing of MR data are provided in Appendix A.1 and Appendix A.2 The synthesis of CE-MRIs via model inference was performed in a Python 3.10.12 environment.

Quantitative assessment of whole-breast similarity between acquired and synthesized CE-MRI included metrics of structural similarity index (SSIM) and normalized neighborhood cross-correlation (CC), along with error metric of normalized root mean square error (NRMSE). SSIM measures perceptual similarity between two images by comparing local structure, intensity (luminance), and contrast. It is designed to reflect how similar the images appear to the human eye. Values range from 0 to 1, with higher values indicating greater similarity. CC measures the degree of linear correlation between corresponding regions in the two images and evaluates how well intensity patterns align spatially. Values range from −1 to 1, with higher values indicating stronger similarity. NRMSE measures the average pixel-wise difference (error) between the two images, normalized to make it scale-independent. Values typically range from 0 to 1, with lower values indicating better agreement.

### 2.3. Tumor Volume Calculation

**Figure A1:**
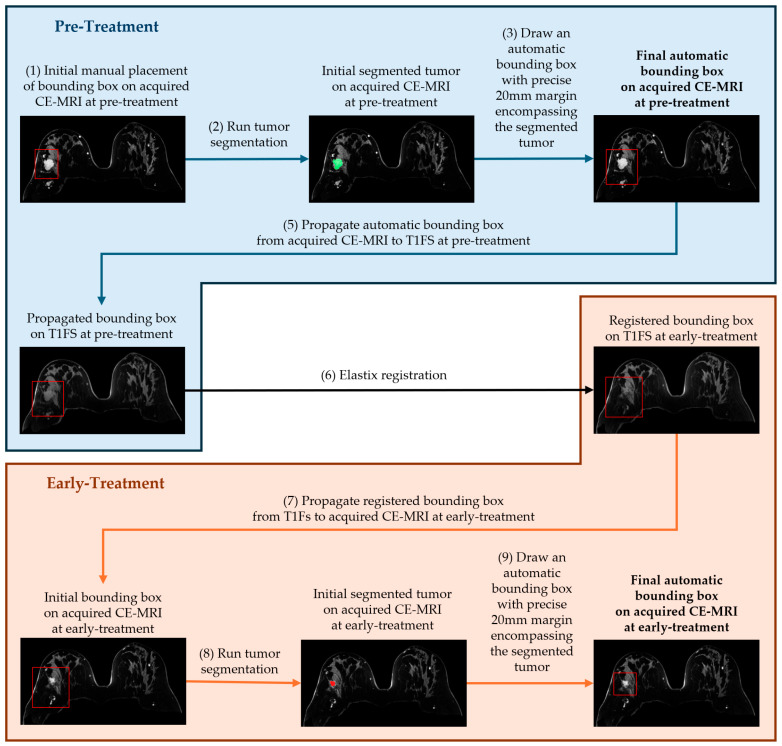
Flowchart outlines the steps for tumor bounding box generation.

In order to reduce operator influence and variability for tumor volume calculation across methods and treatment time points, we used a previously developed semi-automated 3D tumor segmentation workflow (incorporating nnU-Net [24] based DL model [25]) for both acquired and synthesized CE-MRI. A 3D bounding box was first manually defined by an operator to encompass the primary biopsy-proven tumor for each patient at pre-treatment, while avoiding other enhancing regions. The segmentation software tool was then applied to automatically segment the enhancing tumor region within the bounding box. Resulting segmentations and propagated bounding boxes across serial examinations were visually reviewed for adequate tumor coverage and anatomical consistency, with manual adjustment performed when necessary. Tumor volume was calculated based on segmented tumor regions (number of voxels multiplied by voxel size) at baseline and each treatment time point. Details and process flowchart of sequential tumor bounding box generation are provided in Appendix B.1 (Figure A1). Although the segmentation approach reduced manual contouring requirements, the workflow retained operator dependence through initial tumor localization, visual quality review, and occasional bounding box adjustment. Inter-reader variability and reproducibility across serial examinations were not formally evaluated in this pilot study. The tumor segmentation workflow was conducted in a Python 3.9.21 environment.

### 2.4. Statistical Analysis

#### 2.4.1. Agreement of Synthetic with Acquired CE Tumor Segmentations

Dice scores, precision, and recall were computed between synthetic and acquired CE-MRI 3D tumor segmentations using Equations (1)–(3) to quantify spatial overlap and detection accuracy, with acquired CE tumor segmentation as ground truth:

(1) $$Dice=\frac{2\times TP}{2\times TP+FP+FN}$$

(2) $$Precision=\frac{TP}{TP+FP}$$

(3) $$Recall=\frac{TP}{TP+FN}$$

where TP = true positives, representing tumor voxels correctly identified by synthetic CE-MRI segmentations (i.e., overlapping with acquired CE-MRI tumor regions); FP = false positives, representing voxels identified as tumor in the synthetic segmentation that are not present in the acquired tumor segmentation; FN = false negatives, representing tumor voxels present in the acquired segmentation that are not detected in the synthetic CE-MRI segmentation. Dice score quantifies the overall spatial overlap (localization and extent) between the synthetic and acquired tumor segmentations, balancing both precision and recall. Higher values indicate greater agreement between the two segmentations. Precision quantifies the proportion of voxels identified as tumor in the synthetic segmentation that are correctly matched to the acquired tumor segmentation, reflecting the reliability of predicted tumor regions. Recall measures the proportion of tumor voxels in the acquired segmentation that are successfully detected in the synthetic segmentation, reflecting the completeness of tumor detection.

#### 2.4.2. Saliency Score Analysis

Saliency analyses were performed during inference of the synthetic CE-MRI DL model [16], without retraining or patient-specific optimization, to assess model attention patterns associated with each of the six non-contrast inputs to the segmented synthetic tumor region. Saliency maps (generated in Python TensorFlow, details provided in Appendix B.2) were first computed within the full tumor bounding box and normalized within this region to ensure a consistent scale across input channels. The analysis was then restricted to the segmented synthetic tumor region, which represents the model-predicted enhancing tumor area. Within this region, the absolute saliency values (i.e., magnitude, ignoring sign) were summed for each input channel. Each input’s influence (i.e., how sensitive the model output is to changes in the input) was expressed as a percentage of the total saliency across all inputs, and inputs were ranked according to their relative influence on the synthetic tumor volume. Saliency analysis reflects the sensitivity of model output to input perturbations and should not be interpreted as direct evidence of causal feature importance or biological relevance.

#### 2.4.3. Tumor Volume Analysis

The relationship between acquired and synthetic tumor volume was evaluated using Spearman’s correlation coefficient (ρ) at pre-treatment, early treatment, and mid-treatment. Agreement and bias between the two methods were assessed using Bland–Altman plots. The percent decrease in tumor volume from baseline (pre-treatment) was calculated for early and mid-treatment MRIs. Performance in predicting treatment outcome of RCB class 0 and combined 0/1 based on percent decreases in tumor volume was evaluated using AUC-ROC curves. Confidence intervals (95%) for AUCs were estimated using bootstrap resampling (2000 iterations), and differences in AUCs were compared with DeLong’s test.

All statistical analyses were performed in Python (version 3.12.4). Given the exploratory and hypothesis-generating nature of this pilot study and the limited sample size, statistical analyses were primarily intended for descriptive and comparative assessment rather than confirmatory inference. No formal adjustment for multiple comparisons was applied; a *p* value < 0.05 was considered significant.

## 3. Results

### 3.1. Patient Characteristics

Patient clinical and tumor characteristics are summarized in Table 1 and Table 2, respectively. For the 27 patients included in the study, ages ranged from 28 to 75 (median, 47) years, and most were premenopausal (56%) with heterogeneous or extremely dense breasts (70%). Cancers were predominantly invasive ductal carcinomas (96%), high grade (70% Grade 3), ER-positive (59%), and HER2-negative (74%). Favorable RCB treatment outcomes were observed in 14/27 (52%) patients with RCB 0 (pCR) and 6/27 (22%) patients with RCB 1 (minimal residual disease). Mid-treatment analyses included 26 patients due to one missing mid-treatment MRI.

### 3.2. Whole-Breast Similarity and Error Metrics Assessment

**Figure 1:**
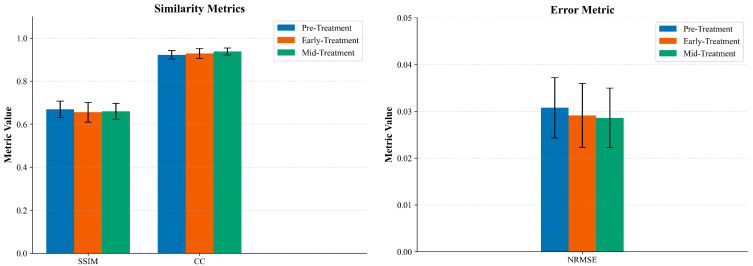
Bar chart showing quantitative similarity (structural similarity index [SSIM] and normalized neighborhood cross-correlation [CC]) and error metrics (normalized root mean square error [NRMSE]) for acquired vs. synthesized CE-MRI at pre-treatment, early treatment, and mid-treatment. Data is shown for 27 patients at pre- and early treatment, and 26 patients at mid-treatment (one did not undergo the mid-treatment MRI).

**Figure 2:**
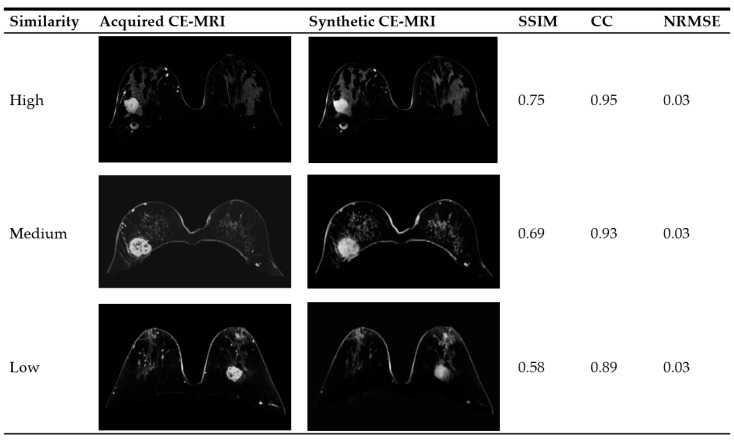
Examples of acquired and synthetic CE-MRI pairs in three study patients. Images shown are actual post-contrast fat-suppressed T1-weighted images (acquired), where invasive breast tumors are evident as brightly enhanced masses, and corresponding synthesized CE-MRI representations, where the tumors also appear bright. Examples shown exibit high, medium, and low similarity (structural similarity index [SSIM] and normalized neighborhood cross-correlation [CC]), despite identical normalized root mean square error (NRMSE) across all three cases.

The similarities and errors between each pair of acquired vs. synthetic CE-MRI were assessed for the full 3D image volumes at each treatment time point (Figure 1). At pre-treatment, similarity metrics demonstrated acceptable-to-strong similarity between acquired and synthesized CE-MRI. SSIM 0.67 ± 0.04 indicates acceptable structural agreement, including edges, textures, and anatomical structures. CC 0.92 ± 0.02 indicates strong intensity agreement. In terms of the error metric, NRMSE 0.03 ± 0.01 demonstrates very low global intensity error. All three metrics remained in similar ranges for pre-treatment, early treatment, and mid-treatment exams, with less than 2% difference in SSIM and CC, and less than 7% difference in NRMSE, suggesting consistent model performance in synthesizing CE-MRI at pre-treatment, early treatment, and mid-treatment. Examples of acquired and synthetic CE-MRI with high, medium, and low similarity (as measured by SSIM and CC), while exhibiting identical NRMSE across all three cases, are shown in Figure 2.

### 3.3. Agreement of Tumor Segmentations

**Figure 3:**
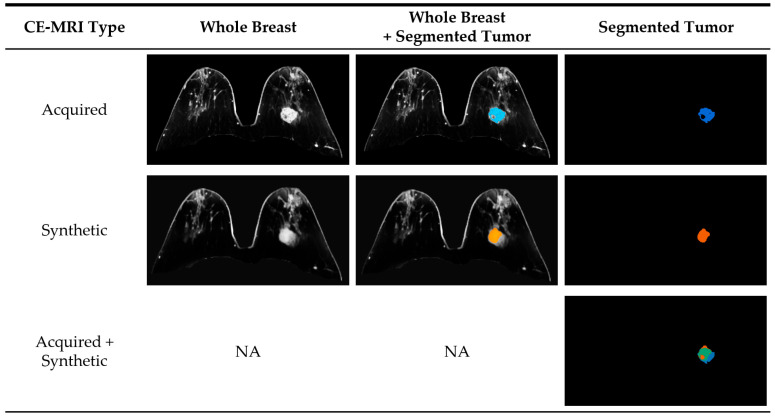
Segmented tumor on acquired and synthetic CE-MRI for an example case, illustrating typical lesion-level mismatch. Shown are representative post-contrast fat-suppressed T1-weighted images from acquired CE-MRI (**left column**, **top row**) and corresponding synthesized CE-MRI (**left column**, **middle row**) at pre-treatment, where a tumor appears bright. Segmented tumor regions are shown in color: acquired tumor (blue) and synthetic tumor (orange). On the combined map (**bottom row**), overlap between the acquired and synthetic tumor (green) corresponds to true-positive (TP) voxels, synthetic only (orange) corresponds to false-positive (FP) voxels, and acquired only (blue) corresponds to false-negative (FN) voxels.

Next, we evaluated the accuracy of synthetic CE-MRI for specifically characterizing tumor extent. Spatial overlap (Dice) and detection accuracy (precision and recall) of the automatically segmented tumor regions between acquired and synthesized CE-MRI are shown in Table 3. At pre-treatment, moderate overlap was observed between acquired and synthesized tumor regions, with a mean ± SD Dice coefficient of 0.57 ± 0.22, moderate precision of 0.70 ± 0.27, and low recall of 0.50 ± 0.22, indicating incomplete lesion-level agreement, with synthetic segmentation capturing only a subset of the acquired tumor extent at baseline, but still missing a large portion of the acquired CE-MRI tumor. At early treatment, Dice, precision, and recall decreased from pre-treatment, indicating substantially reduced lesion-level spatial agreement compared to baseline (Dice = 0.40 ± 0.26), substantial over-segmentation with higher false positives (precision = 0.44 ± 0.30), and substantial under-segmentation with higher false negatives (recall = 0.44 ± 0.29). At mid-treatment, Dice, precision, and recall decreased even further, indicating poor and clinically unreliable lesion-level segmentation agreement at mid-treatment. An example case illustrating segmented tumor regions on pre-treatment acquired and synthetic CE-MRI with Dice = 0.59, precision = 0.64, and recall = 0.55—values close to the cohort’s averages—is shown in Figure 3. Overall, segmentation performance showed a progressive decline from pre-treatment to mid-treatment across all metrics, indicating reduced lesion-level agreement during treatment.

### 3.4. Saliency Score Assessment

**Figure 4:**
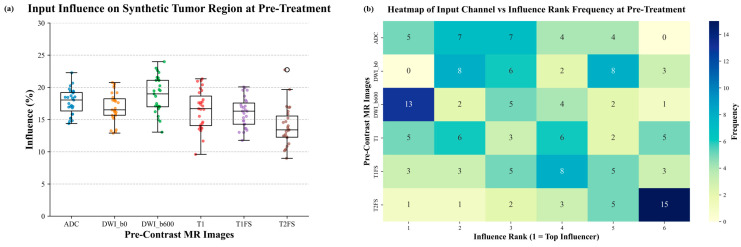
Saliency-based influence analysis of input image types (ADC, DWI_b0, DWI_b600, T1, T1FS, and T2FS) for synthetic CE-MRI tumor enhancement at pre-treatment. (**a**) box plots of percentage of influence of each input channel (in different colors), with boxes showing IQR, center lines indicating medians, whiskers showing data range, and colored circles representing individual patient values. (**b**) heatmap of influence rank (1 = top influencer) frequency, where darker colors indicate higher frequency.

**Figure A2:**
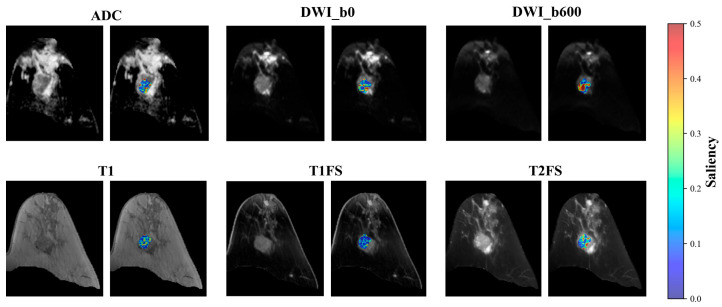
Saliency map (in color) of the synthetic tumor region overlaid on six non-contrast input images to the synthetic CE-MRI DL model, shown for the case in Figure 3 (left breast).

**Figure A3:**
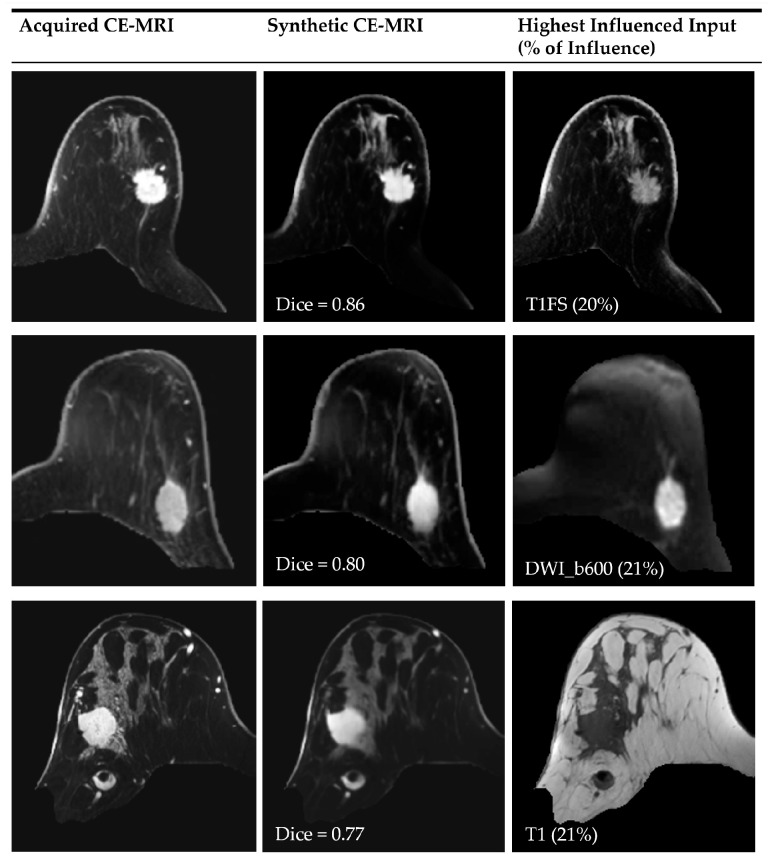
Examples from three patients with differing primary influencer images on the synthetic tumor volume. Shown are pre-treatment acquired CE-MRI, synthetic CE-MRI, their corresponding tumor volume Dice coefficients, and the non-contrast MR inputs with the strongest influence on synthetic tumor volume.

Saliency scores, representing the percentage influence of each input channel on the synthetic enhanced tumor region at pre-treatment, are shown for all tumors in Figure 4a. DWI_b600 had the highest average influence (mean ± SD: 19% ± 3%), followed by ADC (18% ± 2%), DWI_b0 (17% ± 2%), T1 (17% ± 3%), T1FS (16% ± 2%), and T2FS, which had the lowest average influence (14% ± 3%). The frequency of saliency-based ranking (with rank 1 indicating the highest influencer) for each input channel at pre-treatment is shown in Figure 4b. DWI_b600 was most frequently ranked as the top influencer, for 13 of 27 patients, followed by ADC and T1 (five patients each), T1FS (three patients), and T2FS (one patient), while DWI_b0 was never ranked as the top influencer. A corresponding saliency map from the case in Figure 3 is shown in Figure A2 (Appendix B.2). Representative cases in which different input image types are identified as the primary influencers to the saliency scores are shown in Figure A3 (Appendix B.2).

**Figure 5:**
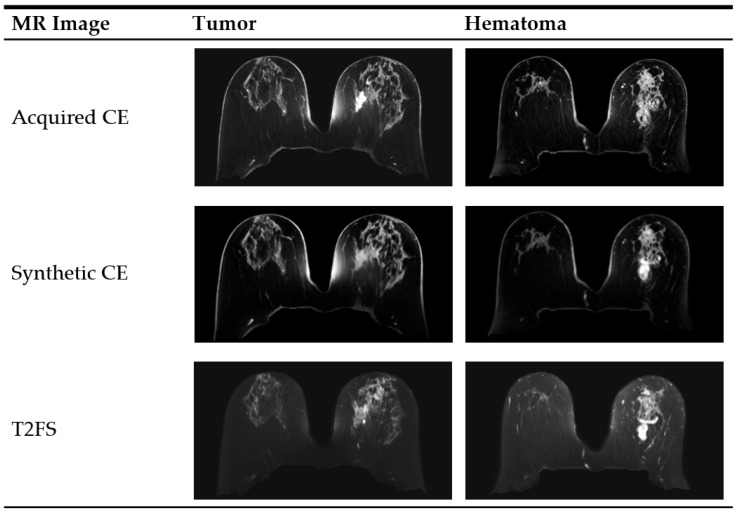
Example synthetic CE-MRI false-negative tumor volume. Axial-view of acquired CE-MRI, synthetic CE-MRI, and non-contrast acquired T2-weighted with fat suppression (T2FS) of tumor and hematoma slices of a patient with no overlap between acquired and synthetic tumor volume at pre-treatment (Dice coefficient = 0.0).

One case resulted in a Dice coefficient = 0.0 at pre-treatment. Visual inspection showed the absence of synthetic enhancement within the true tumor region (false negative), resulting in zero tumor volume (Figure 5). In this case, the model instead generated an enhancement in a separate hematoma region. The falsely enhanced region on synthetic CE-MRI spatially corresponded to a hyperintense area on the T2FS image, suggesting that non-tumor tissue characteristics contributed to the erroneous enhancement pattern.

### 3.5. Tumor Volume Assessment

**Figure 6:**
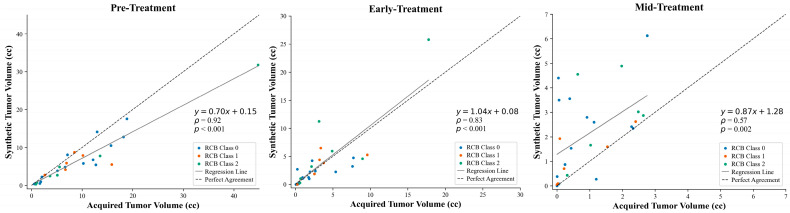
Scatter plot shows tumor volume assessment in cc between acquired and synthesized CE-MRI at pre-treatment (*n* = 27), early treatment (*n* = 27), and mid-treatment (*n* = 26). Dotted line represents line of unity, perfect agreement.

Comparisons of tumor volume (cc) between acquired and synthesized CE-MRI at pre-treatment, early treatment, and mid-treatment are shown in Figure 6. Synthetic tumor volume demonstrated a very strong correlation with acquired tumor volume at pre-treatment (ρ = 0.92, *p* < 0.001), strong correlation at early treatment (ρ = 0.83, *p* < 0.001), and moderate correlation at mid-treatment (ρ = 0.57, *p* = 0.002). Agreement between the two methods was further assessed using Bland–Altman plots, shown in Section Tumor Volume Agreement Using Bland–Altman Plots. Compared to acquired CE-MRI tumor volume measures, synthetic tumor volumes tended to underestimate lesion size at pre-treatment, particularly for larger tumors > 10 cc (mean difference = −2.64 cc), while they most always overestimated lesion size at mid-treatment (mean difference = 1.16 cc), resulting in false-positive tumor volume in some cases with minimal residual tumor.

### 3.6. Prediction of Residual Cancer Burden (RCB) Treatment Outcome

**Figure 7:**
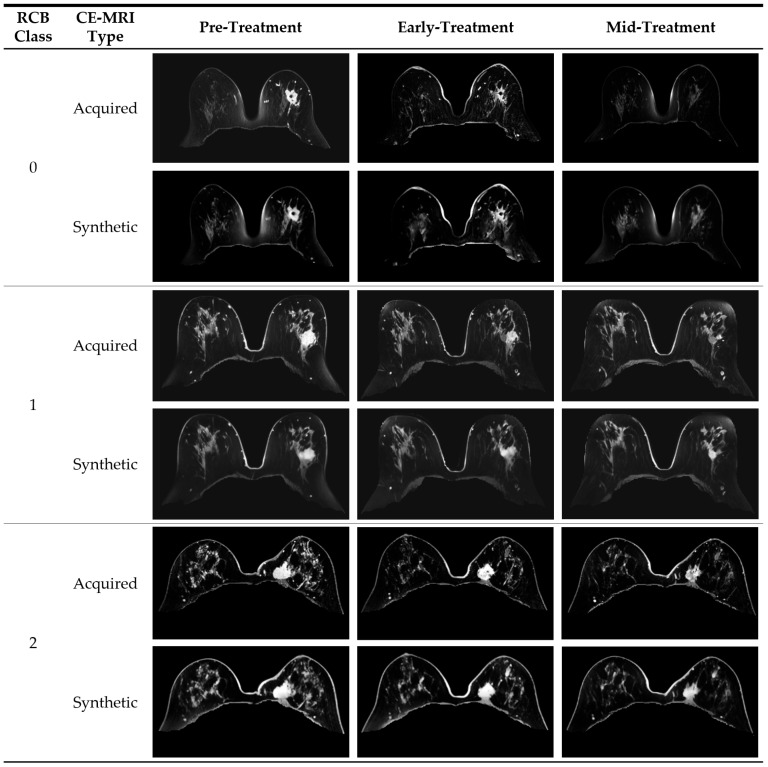
Example axial-view acquired vs. synthetic CE-MRI at pre-treatment, early treatment, and mid-treatment for study patients with treatment outcomes of RCB class 0, 1, and 2. Images shown are post-contrast fat-suppressed T1-weighted images (acquired) and corresponding synthesized CE-MRI representations, where the tumors are each bright at pre-treatment, and exhibit varying degrees of enhancement at early and mid-treatment. Each pair of images (acquired and synthetic) are shown at the same spatial location through the central portion of the tumor. Top: 53-year-old woman with a Grade 3 invasive ductal carcinoma, ER−/PR−/HER2+, Ki-67 not available, with RCB class 0 outcome to NAT. Middle: 63-year-old woman with a Grade 3 invasive ductal carcinoma, ER−/PR−/HER2−, Ki-67 50% with RCB class 1 outcome to NAT. Bottom: 49-year-old woman with a Grade 1 invasive ductal carcinoma, with ER+/PR+/HER2−, Ki-67 15%, with RCB class 2 outcome to NAT.

**Figure 8:**
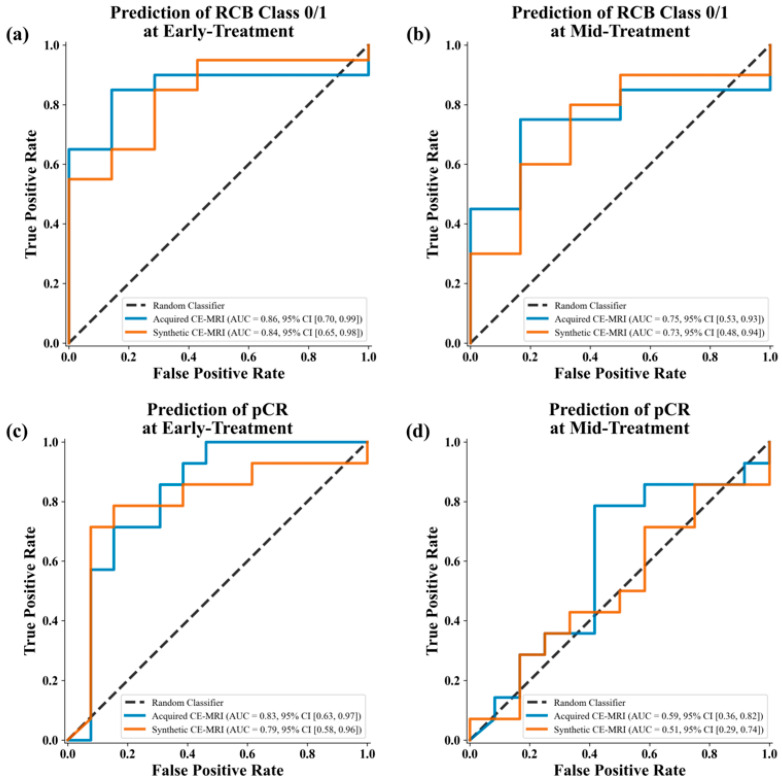
ROC curves of percent decrease in tumor volume in predicting (**a**) RCB class 0/1 (*n* = 20) vs. RCB class 2/3 (*n* = 7) at early treatment, (**b**) RCB class 0/1 vs. RCB class 2/3 (*n* = 6 *) at mid-treatment, (**c**) pCR (RCB class 0, *n* = 14) vs. non-pCR (RCB class 1/2/3, *n* = 13) at early treatment, and (**d**) pCR (RCB class 0, *n* = 14) vs. non-pCR (RCB class 1/2/3, *n* = 12 *) at mid-treatment. * Note: One patient with RCB class 2 outcome did not undergo mid-treatment MRI.

Examples of acquired vs. synthetic CE-MRIs for patients with RCB class 0, 1, and 2 treatment outcomes are shown in Figure 7. AUC-ROC curves of acquired vs. synthesized CE-MRI in predicting RCB class 0/1 vs. 2/3 and RCB class 0 (pCR) vs. 1/2/3 using percent decrease in tumor volume at early treatment and mid-treatment are shown in Figure 8. Percent decrease in synthetic tumor volume demonstrated strong performance in predicting RCB class 0/1 (AUC = 0.84, 95% CI [0.65, 0.98]) at early treatment, comparable to that of acquired tumor volume (AUC = 0.86, 95% CI [0.70, 0.99]; *p* = 0.83). At mid-treatment, predictive performance for RCB class 0/1 numerically decreased relative to early treatment for both synthetic and acquired tumor volume (AUC = 0.73, 95% CI [0.48, 0.94] and AUC = 0.75, 95% CI [0.53, 0.93], respectively), suggesting reduced discrimination compared with early treatment; however, predictive performance remained similar between synthetic and acquired CE-MRI at mid-treatment (*p* = 0.80). Similarly, performance for predicting RCB class 0 vs. 1/2/3 declined from early treatment to mid-treatment for both synthetic and acquired tumor volumes (Figure 8), with no statistically significant differences between methods at either time point (*p* = 0.70 and *p* = 0.26, respectively).

## 4. Discussion

Our study evaluated the application of a previously developed U-Net-based DL model for synthesizing breast CE-MRI from non-contrast MRI inputs [16] and monitoring preoperative therapy in an independent cohort without additional retraining or fine-tuning. The primary focus was on the fidelity of the synthesized images and the accuracy of measuring treatment-induced changes in tumor volume. Across pre-treatment, early treatment, and mid-treatment time points, the results demonstrated that synthetic CE-MRI achieved acceptable agreement with acquired CE-MRI in terms of anatomical structure and signal intensity of the whole breast, supporting its preliminary feasibility as a non-contrast-derived surrogate representation of CE-MRI at the whole-breast level, while lesion-level agreement was more limited and decreased during treatment. Because the model was originally trained using data from a separate institution, these findings also provide preliminary insight into the feasibility of applying synthetic CE-MRI approaches across datasets acquired under different institutional and imaging conditions, although the effects of domain shift remain incompletely characterized. From a secondary clinical perspective, exploratory analyses suggested similar trends between synthetic and acquired CE-MRI for early identification of response to NAT, particularly in patients who ultimately achieved RCB class 0/1 outcomes, although synthetic CE-MRI did not provide fully accurate absolute tumor volume estimates relative to acquired CE-MRI. Additional training of the synthetic CE-MRI model to improve anatomical similarity (e.g., SSIM ≥ 0.90) may further enhance its performance for quantitative tumor characterization.

At pre-treatment, synthetic CE-MRI mildly underestimated tumor volume, as the model commonly enhanced only a portion of the tumor region evident on acquired CE-MRI. This underestimation may be related to the model’s reliance on specific input modalities in defining enhancement patterns. Notably, nearly half of the study cohort (13/27 patients) exhibited DWI_b600 as the primary influencer of the synthetically enhanced tumor regions at pre-treatment. This finding was based on saliency score analysis computed within the segmented synthetic tumor region, which reflects voxel-wise sensitivity of the synthesized tumor enhancement to variation in each input channel. Importantly, this approach differs from the ablation-based analysis in the original model development study, which systematically removed input information and evaluated changes in similarity and error metrics over the entire breast. As a result, saliency-based analysis reflects localized model attention patterns on the synthesized tumor region, whereas ablation analysis captures global changes in reconstruction performance, which may lead to different interpretations regarding the relative importance of DWI_b600. Overall, our findings suggested that diffusion-weighted imaging features were frequently associated with higher saliency responses within the synthesized tumor regions. Furthermore, saliency outputs may be influenced by model architecture, preprocessing methods, normalization strategies, and correlations among imaging inputs. Therefore, the observed prominence of DWI_b600 input should not be interpreted as definitive evidence of biological or mechanistic relevance to synthetic enhancement generation. One possible explanation is that tumor-associated diffusion restriction [26] may produce imaging characteristics that are strongly represented within the learned feature space of the model, although this interpretation remains speculative. These observations are hypothesis-generating and require validation using ablation studies and larger multi-institutional external cohorts acquired across diverse imaging protocols and scanner platforms.

At early treatment, synthetic CE-MRI continued to underestimate tumor volume in comparison with acquired CE-MRI, despite strong correlations between the two. Agreement of tumor segmentation (Dice, precision, and recall) also worsened compared to the pre-treatment setting, possibly due to NAT-induced biological changes, which may reduce the ability of the externally trained synthesis model to generalize across evolving tumor appearances during treatment. From early to mid-treatment, Dice, precision, and recall decreased significantly, indicating increasingly unreliable tumor segmentation, with synthetic CE-MRI commonly overestimating residual tumor volume. These findings suggest that lesion-level segmentation performance deteriorates over the course of treatment, even when the relative changes in tumor volume remain relatively preserved.

Although synthetic CE-MRI may not provide sufficiently precise tumor extent measurements for absolute, stand-alone assessment, we evaluated two related but distinct endpoints: (1) favorable pathological response (RCB class 0/1) and (2) pathologic complete response (pCR; RCB class 0). For RCB class 0/1 vs. 2/3, our exploratory analyses suggested that early treatment changes in tumor volume derived from synthetic CE-MRI images provided numerically similar performance to those derived from acquired CE-MRI for predicting RCB class 0/1 pathologic response (synthetic AUC = 0.84 vs. acquired AUC = 0.86; *p* = 0.83). At mid-treatment, predictive performance for RCB class 0/1 decreased for both synthetic and acquired tumor volumes (AUC = 0.73 and 0.75, respectively), although no significant difference was observed between methods (*p* = 0.80). In contrast, for prediction of pCR (RCB class 0) alone, performance was lower than for RCB class 0/1 at both time points and declined from early- to mid-treatment for both synthetic and acquired tumor volumes (early treatment: AUC = 0.79 vs. 0.83; mid-treatment: AUC = 0.51 vs. 0.59), with no statistically significant differences between methods (*p* = 0.70 and *p* = 0.26, respectively). The relatively lower AUCs for pCR prediction, particularly at mid-treatment, may be partly explained by the presence of RCB class 1 cases, where residual tumors are minimal or nearly resolved on imaging, resulting in appearances that can mimic complete response and reduce discriminability. Despite these differences in absolute tumor measurements, the predictive trends observed for both synthetic and acquired CE-MRI are broadly consistent with findings from a large multicenter study, which reported that acquired CE-MRI-derived functional tumor volume (FTV) change is a useful marker of pathologic response at both early treatment (pCR AUC = 0.70; RCB 0/1 AUC = 0.66) and mid-treatment (pCR AUC = 0.72; RCB 0/1 AUC = 0.69) [13].

More broadly, these findings suggest that information relevant to contrast enhancement and treatment response may already be partially encoded within standard non-contrast MRI sequences. Thus, synthetic CE-MRI may function as an intermediate representation that facilitates the interpretation of these latent imaging features. However, determining whether these latent imaging relationships remain stable across institutions, scanner vendors, acquisition protocols, and diverse breast cancer phenotypes requires further investigation. Future work could explore whether similar predictive performance can be achieved by directly modeling treatment response from multi-parametric non-contrast imaging, without explicitly generating synthetic CE-MRI.

This pilot study has limitations that may affect the generalizability of the results to larger populations and model predictive performance. First, the cohort size was small (27 patients in total), with a high rate of patients achieving pCR treatment outcomes (52%), and patients with implants or NMEs were excluded. Exclusion of NME lesions and patients with implants may limit generalizability and may introduce selection bias toward tumors more amenable to synthesis and segmentation. One patient with RCB class 2 outcome did not undergo the mid-treatment MRI, which could also bias mid-treatment findings within this small exploratory cohort (*n* = 26 at mid-treatment). No imputation was performed. Second, the synthetic CE-MRI model was previously developed using a relatively small cohort (96 patients), applied strict exclusion criteria, and lacked external validation [16]. A more robust evaluation could be performed by applying the methodology proposed in this study to a larger cohort and using a more generalized model. Third, tumor volume derived from the early-phase post-contrast image alone is less predictive than FTV for RCB outcomes [27,28]. The synthetic CE-MRI model we explored only generates the single early-phase post-contrast time point and therefore lacks the dynamic information required to calculate FTV. Using a model capable of synthesizing multiple post-contrast time points could potentially address this limitation. Fourth, the automated tumor segmentation tool sometimes produced false positives when the tumor became very small or disappeared, or when no enhancement was present on synthetic CE-MRI. A more robust tumor segmentation algorithm capable of handling negative cases or better accounting for synthetic intensity scaling may help address this limitation. Additionally, the workflow was not fully automated because manual tumor localization, visual quality review, and occasional bounding box adjustment were required. Inter-reader reproducibility and segmentation variability across serial examinations were not formally assessed and warrant evaluation in future studies. Overall, these findings should be interpreted in the context of a proof-of-concept retrospective pilot study and are hypothesis-generating rather than indicative of clinical implementation readiness.

## 5. Conclusions

Synthetic CE-MRI derived from non-contrast MRI demonstrates preliminary feasibility for reproducing whole-breast imaging characteristics and supporting exploratory treatment-response assessment in a proof-of-concept retrospective pilot cohort, especially to identify responders at early treatment (after one cycle of NAT) who will likely achieve RCB class 0/1 at post-treatment. In contrast, prediction of pathologic complete response (pCR; RCB class 0) was less reliable and more variable across treatment time points. This likely reflects the imaging similarity between complete response (RCB 0) and near-complete response (RCB 1), which makes these two categories more difficult to distinguish. These findings suggested that synthetic CE-MRI may be better suited for broader response prediction (RCB 0/1) than for precise identification of pCR. Lesion-level tumor quantification was variable, particularly during treatment, and performance decreased at later time points. The strongest influencing inputs for tumor enhancement accuracy on pre-treatment synthetic CE-MRI were b = 600 s/mm^2^ DW images, followed by T1-weighted images without fat suppression and ADC maps, although these findings should be interpreted cautiously and are not indicative of causal relationships. More refinements are still needed for the synthetic CE-MRI model to overcome challenges related to the presence of hematomas and artifacts on non-contrast images to improve the accuracy of tumor volume measurements and performance for predicting breast cancer response to NAT. Further validation in larger, more heterogeneous multi-institutional cohorts and refinement of synthesis and segmentation pipelines are required before any clinical translation can be considered.

## Figures and Tables

**Table 1:** Patient clinical characteristics.

Characteristic	No. of Women (*n* = 27)
Age (years)	
Median (Range)	47 (28–75)
Menopausal status	
Premenopausal	15 (56%)
Perimenopausal	1 (4%)
Postmenopausal	9 (33%)
Unknown	2 (7%)
Fibroglandular tissue	
Fatty	0 (0%)
Scattered	8 (30%)
Heterogenously dense	16 (59%)
Extremely dense	3 (11%)
Background parenchymal enhancement	
Minimal	6 (22%)
Mild	9 (33%)
Moderate	8 (30%)
Marked	4 (15%)
RCB class	
0	14 (52%)
1	6 (22%)
2	7 (26%)
3	0 (0%)

Note.—Unless otherwise specified, data are numbers of women with percentages in parentheses.

**Table 2:** Primary tumor characteristics.

Characteristic	No. of Women (*n* = 27)
Type of Invasive Breast Cancer	
Invasive Ductal Carcinoma (IDC)	26 (96%)
Invasive Lobular Carcinoma (ILC)	1 (4%)
Estrogen receptor status	
Positive	16 (59%)
Negative	11 (41%)
Progesterone receptor status	
Positive	11 (41%)
Negative	16 (59%)
HER2 status	
Positive	7 (26%)
Negative	20 (74%)
Ki-67 status	
<14%	2 (7%)
≥14%	17 (63%)
Not available	8 (30%)
Nottingham grade	
1	1 (4%)
2	6 (22%)
3	19 (70%)
Not available	1 (4%)
Lesion size (cm)	
Median (Range)	3.6 (1.6–8.8)

Note.—Unless otherwise specified, data are numbers of women with percentages in parentheses.

**Table 3:** Comparison of segmented tumor regions between acquired and synthesized CE-MRI.

Performance Metric	Pre-Treatment (*n* = 27)	Early Treatment (*n* = 27)	Mid-Treatment (*n* = 26)
Dice	0.57 ± 0.22 (0.00–0.86)	0.40 ± 0.26 (0.00–0.75)	0.23 ± 0.27 (0.00–0.74)
Precision	0.70 ± 0.27 (0.00–0.97)	0.44 ± 0.30 (0.00–0.89)	0.20 ± 0.25 (0.00–0.71)
Recall	0.50 ± 0.22 (0.00–0.92)	0.44 ± 0.29 (0.00–0.98)	0.34 ± 0.34 (0.00–0.82)

Note.—Performance metric is reported as the mean ± SD (range).

**Figure A4:**
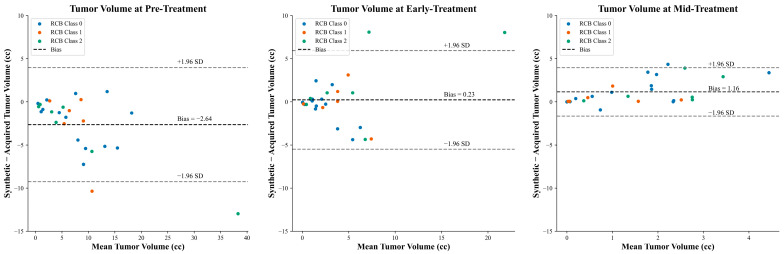
Bland–Altman plot shows tumor volume assessment in cc between acquired and synthesized CE-MRI at pre-treatment, early treatment, and mid-treatment.

## Data Availability

The data presented in this study are available on request from the corresponding author due to privacy restrictions around protected health information.

## Abbreviations

The following abbreviations are used in this manuscript:

ADC	Apparent diffusion coefficient
CE	Contrast-enhanced
DL	Deep learning
DWI_b0	Diffusion-weighted imaging with b = 0 s/mm^2^
DWI_b600	Diffusion-weighted imaging with b = 600 s/mm^2^
FOV	Field of view
MRI	Magnetic resonance imaging
pCR	Pathologic complete response
RCB	Residual cancer burden
T1	T1-weighted without fat suppression
T1FS	T1-weighted with fat suppression
T2FS	T2-weighted with fat suppression

## Appendix A

### Appendix A.1. MRI Acquisition

Bilateral breast MRI examinations, including dynamic contrast-enhanced (DCE) MRI, were performed on 3.0-Tesla scanners (Achieva or Ingenia, Philips Healthcare, Amsterdam, the Netherlands; Signa Premier, GE Healthcare, Chicago, IL, USA). MRI protocol parameters are listed in Table A1.

**Table A1:** Standardized MRI acquisition parameters.

Parameters	DCE/T1FS *	T2FS	T1	DWI
Sequence type	GRE (4D TRAK XD/DISCO)	TSE/FSE	FFE/SPGR	Single shot EPI
2D or 3D sequence	3D	2D	3D	2D
Slice orientation	Axial	Axial	Axial	Axial
Laterality	Bilateral	Bilateral	Bilateral	Bilateral
Phase-encode direction	R/L	R/L	R/L	A/P
Field of view (mm)	240 × 360	240 × 360	240 × 360	360 × 360
Fat suppression	Yes	Yes	No	Yes
Flip angle	10	90	10	90
Acquired in-plane resolution (mm)	1	1	1	1.8
Slice thickness (mm)	1.5	3	1.5	4
Slice gap (mm)	No gap	No gap	No gap	No gap
No. of slices	113	60	113	40
Parallel imaging factor	3	3	3	2.8
TR (ms)	5–7	5500	Shortest	≥3500
TE (ms)	minimum	60	minimum	minimum
EPI factor	–	–	–	66
b values	–	–	–	0, 100, 600, 800
No. of averages	1	1	1	2 (b < 500 s/mm^2^), 4 (b = 500–800 s/mm^2^)
Contrast injection	Intravenous injection of 0.1 mmol/kg body-weight gadoteridol	N/A	N/A	N/A
Dynamic scans	Approx. 34 phases, 17 s per phase (inject contrast after second phase)	N/A	N/A	N/A
Acquisition time (minutes)	≤10:00	2:30	1:20	2:30

* T1FS = T1-weighted images with fat suppression were acquired as the pre-contrast (baseline) first phase images of the DCE series. Abbreviations: DCE, dynamic contrast-enhanced; T2FS, T2-weighted with fat suppression; T1, T1-weighted without fat suppression; T1FS, T1-weighted with fat suppression; DWI, diffusion-weighted imaging; GRE, gradient recalled echo; TSE, turbo spin echo; FSE, fast spin echo; FFE, fast field echo; SPGR, spoiled gradient recalled; EPI, echo planar imaging; TRAK, time-resolved MR angiography with keyhole; DISCO, Differential Subsampling with Cartesian Ordering.

### Appendix A.2. MRI Preprocessing

The MR images obtained from MRI scanners as DICOM files were deidentified using the Radiological Society of North America (RSNA) Clinical Trial Processor (CTP). Initial preprocessing was performed using MATLAB R2024a (MathWorks, Natick, MA, USA). DICOM files were converted into .mat files (MATLAB data files), and the number of slices was truncated to the nearest integer divisible by eight to be compatible with the U-Net-based DL model to synthesize CE-MRI, as non-contrast MR image inputs need to be reduced in size by half three consecutive times (i.e., 2^3^ = 8). The MR images were then motion-corrected (from patients’ movement), interpolated (to ensure all images have the same FOV, in-plane resolution, and slice thickness), and co-registered (to align all images voxel-wise) using MATLAB.

Apparent diffusion coefficient (ADC) maps were generated by voxel-wise fitting of diffusion-weighted images acquired at b-values of 0, 100, 600, and 800 s/mm^2^ to the mono-exponential model of MRI signal decay:

$$S\left(b\right)=S_{0}\cdot e^{-b\cdot ADC}$$

where S(b) is the signal intensity at any given b-value and $S_{0}$ is the signal intensity with no diffusion weighting.

All images were then converted from .mat into NifTI files and resampled into isotropic resolution (1 mm × 1 mm × 1 mm) using Python. The preprocessed T1-weighted fat-suppressed .mat files were also converted into .npy files (numpy array in Python) to be used for segmenting whole-breast with a previously developed DL model [29].

## Appendix B

### Appendix B.1. Tumor Bounding Box Generation

Each tumor bounding box was created using a semi-automated 2-step process, as shown in Figure A1: (1) initial manual placement on an acquired post-contrast image at pre-treatment and (2) automatic bounding box generation. We first manually drew an initial tumor bounding box at pre-treatment to fully encompass the tumor with a generous margin of approximately 20 mm in all dimensions, which was selected to provide sufficient context for the DL model to distinguish the tumor from normal tissue while not overlapping with other lesions (if present). Initial tumor localization was performed using visual assessment of the acquired CE-MRI by the operator. Next, we ran the DL model within this initial bounding box to segment the tumor, producing an irregular tumor mask used for subsequent analysis (e.g., volume estimation). To standardize the region of interest and ensure adequate contextual information for downstream processing, we then programmatically generated a final bounding box by expanding the segmented tumor mask by an additional fixed margin of 20 mm in all dimensions. This second expansion ensures that the final bounding box consistently contains both the tumor and sufficient surrounding tissue, improving the robustness of model performance across cases and time points. Each final pre-treatment tumor bounding box was then translated to early treatment exams using elastix registration [30] to co-register the pre-treatment to post-treatment images and apply the same transformation matrix to the bounding box. Registration steps included rigid, affine, and b-spline transformations of pre-treatment CE-MRI to early treatment CE-MRI. Each resulting transformed bounding box was further reformed into the smallest parallel bounding box completely encompassing the transformed bounding box, representing an initial bounding box at early treatment. The final automatic bounding box was automatically generated in a similar fashion as that of the pre-treatment using the DL model to segment the tumor within this initial bounding box and then adding 20 mm margin in all dimensions. All propagated and automatically generated bounding boxes were visually reviewed to confirm adequate tumor inclusion and anatomical consistency across serial examinations. The bounding box might need to be manually adjusted if the propagated location does not fully encompass the tumor or if there is insufficient context for the segmentation model to operate deterministically based on visual inspection by the operator. Consequently, although segmentation itself was DL-assisted, the overall workflow retained partial operator dependence.

### Appendix B.2. Saliency Score Analysis

Saliency maps provide voxel-wise sensitivity maps that indicate how strongly each voxel in the non-contrast images influences the model’s synthetic CE prediction [31]. These maps were computed by measuring the sensitivity of the model’s output with respect to changes in the input image intensity, i.e., by calculating the gradient of the predicted synthetic CE image with respect to the input non-contrast images after the model inference.

A corresponding saliency map from the case in Figure 3 is shown in Figure A2. DWI_b600 is the highest influencer and shows a greater number of tumor pixels with high saliency scores compared to the other input channels. As shown in the raw DWI_b600 image without saliency overlay, only a subset of the acquired tumor region is highlighted, which corresponds to the enhancing area observed on the synthetic CE-MRI.

Saliency score ranking was used to examine cases with low as well as high quantitative tumor segmentation performance at pre-treatment. Figure A3 shows representative cases in which different input image types are identified as the primary influencers of the saliency scores. Synthetic CE-MRI enhancement corresponded to bright regions on T1FS, and DWI_b600, and dark region T1 image as top input influencers. In the case with the highest Dice score of 0.86, T1FS was the top influencer (20% of influence).

## Appendix C

### Tumor Volume Agreement Using Bland–Altman Plots

Agreement between acquired and synthesized CE-MRI was assessed using Bland–Altman plots, which display the mean difference (bias) and 95% limits of agreement (LoA). Comparisons of tumor volume (cc) between acquired and synthesized CE-MRI at pre-treatment, early treatment, and mid-treatment are shown in Figure A4. At pre-treatment, synthetic tumor volume slightly underestimated acquired tumor volume on average (bias = −2.64 cc, 95% LoA = −9.26 to 3.99 cc), and this difference was statistically significant (paired *t*-test: t = −3.98, *p* < 0.001). At early treatment, synthetic tumor volume minimally overestimated acquired tumor volume (bias = 0.23 cc, 95% LoA = −5.49 to 5.94 cc), with no significant difference (t = 0.40, *p* = 0.692). At mid-treatment, synthetic tumor volume slightly overestimated acquired tumor volume (bias = 1.16 cc, 95% LoA = −1.66 to 3.98 cc), and the difference was statistically significant (t = 4.02, *p* < 0.001).
